# Baseline predictors of short-term visual outcomes after intravitreal conbercept injection for neovascular age-related macular degeneration

**DOI:** 10.5935/0004-2749.20230018

**Published:** 2022-01-31

**Authors:** Peng Zhang, Jing Shi, Lei Gao, Xiang-Wen Shu

**Affiliations:** 1 Department of Ophthalmology, Jinan Second People’s Hospital, Jinan, Shandong Province, China.

**Keywords:** Macular degeneration, Intraviteal injections, Conbercept, Tomography, optical coherence, Macular degeneration, Injeções intravítreas Combercepte, Tomografia de coerência óptica

## Abstract

**Purpose:**

Neovascular age-related macular degeneration is the leading cause of vision loss in the elderly. We aimed to identify baseline predictors of visual prognosis after intravitreal conbercept injection for neovascular age-related macular degeneration.

**Methods:**

We conducted a retrospective review of 58 patients with neovascular age-related macular degeneration who were treated with intravitreal injections of conbercept 0.5 mg in routine clinical practice. Basic information such as age, sex, intraocular pressure, and disease course was collected. Best-corrected visual acuity, mean retinal sensitivity, and optical coherence tomography findings were recorded at baseline and 6 months after treatment. Logistic regression analysis was used to identify independent predictors of best-corrected visual acuity at 6 months after treatment.

**Results:**

After the 6-month treatment, the mean best-corrected visual acuity improved from 1.10 ± 0.42 logarithm of the minimum angle of resolution (logMAR) to 0.41 ± 0.18 logMAR, the mean retinal sensitivity increased from 5.13 ± 0.86 dB to 7.32 ± 1.21 dB, the mean central retinal thickness decreased from 440.38 ± 61.05 µm to 260.01 ± 24.86 µm, and the total number of hyperreflective dots and the number of hyperreflective dots in each retina layer were significantly reduced as compared with those before treatment (all p<0.05). Twenty-two patients showed improved vision, and 36 had unimproved vision. Multivariate analyses revealed that the number of subretinal hyperreflective dots, the state of external limiting membrane, baseline best-corrected visual acuity, and age were independent predictors of best-corrected visual acuity (all p<0.05).

**Conclusion:**

Poor recovery of patients after intravitreal conbercept injection may be related to the number of subretinal hyperreflective dots, the state of external limiting membrane, baseline best-corrected visual acuity, and age, which may be used as predictors of short-term visual outcomes and should be fully evaluated before operation.

## Abstract (pt)

**Objetivo:**

A degeneração macular neovascular relacionada à idade é a principal causa de perda de visão em idosos. O objetivo deste estudo foi identificar os preditores iniciais que afetam o prognóstico visual após a injeção intravítrea de combercepte para degeneração macular neovascular relacionada à idade.

**Métodos:**

Esta é uma revisão retrospectiva de 58 pacientes com degeneração macular neovascular relacionada à idade que foram tratados com injeções intravítreas de 0,5 mg de combercepte na prática clínica de rotina. Foram coletadas informações básicas, tais como idade, sexo, pressão intraocular e evolução da doença. A melhor acuidade visual corrigida, as sensibilidades retinianas médias e varreduras de tomografia de coerência óptica foram registradas no início do estudo e 6 meses após o tratamento. Foi efetuada uma análise de regressão logística para determinar os preditores independentes da melhor acuidade visual corrigida 6 meses após o tratamento.

**Resultados:**

Após 6 meses de tratamento, a média da acuidade visual melhor corrigida melhorou de 1,10 ± 0,42 para 0,41 ± 0,18 logMAR; as sensibilidades retinianas médias aumentaram de 5,13 ± 0,86 para 7,32 ± 1,21 dB; a espessura retiniana central média diminuiu de 440,38 ± 61,05 para 260,01 ± 24,86 µm; e os pontos hiper-reflexivos, tanto em números totais quanto em cada camada de retina, foram significativamente reduzidos em comparação com os valores de antes do tratamento (todos com p<0,05). Houve 22 pacientes com visão melhorada e 36 pacientes com visão não melhorada. As análises multivariadas mostraram que o número de pontos hiper-reflexivos sub-retinianos, o estado da membrana limitante externa, a melhor acuidade visual corrigida inicial e a idade foram preditores independentes para a melhor acuidade visual corrigida (todos com p<0,05).

**Conclusão:**

A má recuperação de pacientes após a injeção de combercepte intravítreo pode estar relacionada ao número de pontos hiper-reflexivos sub-retinianos, ao estado da membrana limitante externa, à acuidade visual corrigida inicial e à idade, parâmetros que podem ser usados como preditores de resultados visuais de curto prazo e devem ser totalmente avaliados antes da cirurgia.

## INTRODUCTION

Age-related macular degeneration (AMD), a progressive chronic disease affecting the central retina, is the leading cause of vision loss worldwide^(1)^. AMD could result in gradual loss and impairment of vision in the elderly, and its prevalence is predicted to increase with aging populations^(2,3)^. AMD can be divided into regional atrophy and neovascular AMD (nAMD), with the latter accounting for 90% of all cases of AMD-related visual loss. The vascular endothelial growth factor (VEGF) plays a key role in the pathogenesis of nAMD owing to its ability to regulate angiogenesis^(4)^. With the introduction of anti-VEGF agents represented by ranibizumab, anti-VEGF drugs have become the first-line treatment for nAMD^(5,6)^. At present, the common anti-VEGF drugs in clinical practice include ranibizumab, aflibercept, and conbercept.

Although anti-VEGF drugs have improved patients’ visions to a certain extent, not all patients with nAMD can achieve improved or maintained vision after treatment in clinical practice. Li et al. found that only 50.0% of patients gained ≥15 letters at 12 months after receiving conbercept treatment^(7)^. Therefore, the prognostic factors of the effect of drug therapy for nAMD must be identified. Previous studies on the visual prognostic factors of anti-VEGF therapy showed that baseline BCVA, the external limiting membrane (ELM), the ellipsoid zone (EZ), hyperreflective dots (HRDs), and other indicators were related factors^(8,9)^. A prognostic factor analysis was performed in 96 patients (61 with polypoidal choroidal vasculopathy and 35 with nAMD) and revealed that baseline BVCA, age, presence of HRDs, and ELM status correlated with final visual acuity in the Pearson’s correlation analyses, while in the multiple regression analysis, only baseline BVCA and ELM status were related to visual prognosis^(10)^. More large-scale research is needed on the prognostic factors of nAMD treated with anti-VEGF drugs.

Conbercept, a recombinant fusion protein developed in China, has a high affinity with all VEGF isoforms and placental growth factor^(11)^. Preclinical studies have demonstrated its anti-angiogenesis activity in both ocular neovascular disease and tumor models^(12,13)^. Clinical trials of conbercept have shown its superior efficacy and safety^(7)^. A recent meta-analysis revealed that conbercept was superior to ranibizumab with respect to visual gain after treatment^(14)^. A systematic review also revealed that conbercept was superior to ranibizumab in reducing central retinal thickness (CRT), lowering the plasma level of VEGF, and safety^(15)^. A cost-effectiveness analysis based on the Markov model concluded that compared with ranibizumab and aflibercept, conbercept was a cost effective alternative treatment for nAMD in a Chinese health-care setting^(4)^.

To the best of our knowledge, only few reports have identified baseline predictors that affect the treatment of nAMD with conbercept. Our study provides a comprehensive predictor analysis of intravitreal conbercept (IVC) injection for nAMD, including age, sex, disease course, baseline BCVA, intraocular pressure (IOP), CRT, number of HRDs, and EZ and ELM statuses, which provide a more accurate assessment of the potential benefits of conbercept treatment and an understanding of the mechanisms of action of these anti-VEGF drugs.

## METHODS

This is a retrospective study of patients treated with IVC injection for nAMD. Ethics approval was obtained from the ethics committee of Jinan Second People’s Hospital. The study adhered to the tenets of the Declaration of Helsinki. Owing to the retrospective nature of the study, written informed consent was not required.

### Patients

The study included 58 patients (58 eyes) who began their first treatment with IVC injection for nAMD between January 2018 and January 2019 at Jinan Second People’s Hospital. Patients with any type of subfoveal or parafoveal choroidal neovascularization (CNV) caused by AMD diagnosed by fundus fluorescein angiography (FFA) and indocyanine green angiography (IGA) who were older than 50 years were included in the study. Furthermore, follow-up for at least 6 months was required.

The exclusion criteria were as follows: 1) patients treated with photodynamic therapy or intravitreal anti-VEGF drugs; 2) patients with diabetic retinopathy, polypoidal vasculopathy, or other retinal diseases; 3) patients with a history of internal surgery other than cataract; 4) patients with a severe systemic disease that could affect the outcome of intravitreal injection; 5) patients with CNV due to other causes; 6) patients who could not undergo fundus examination because of unclear optical media.

### Treatment protocol

The 3+pro re nata (injection once a month for three consecutive months and then reinjection as needed) regimen were adopted. All the eyes were treated with intravitreal injection of 0.5 mg/0.05 mL conbercept (Chengdu Kang Hong Biotech Co, Ltd, Sichuan, China) by the same physician. All the patients received levofloxacin eye drops at 0.5% for 3 consecutive days (4 times per day) before and after IVC injection. Reinjection was performed if any intraretinal or subretinal fluid was observed on optical coherence tomography (OCT).

### Data collection

At baseline, BCVA examination, IOP measurement, and fundoscopy were performed. The international standard visual acuity chart was used for BCVA examination, which was converted into logarithm of the minimum angle of resolution (logMAR) in the statistical calculation. Noncontact tonometer (Topcon, Tokyo, Japan) was used for the IOP measurement, and fundus photochromy (FP) detection was performed using products manufactured by a company in Heidelberg, Germany.

Microperimetry was performed for all the patients using the MP-1 Microperimeter (Nidek Technologies, Padova, Italy). The mean retinal sensitivity were calculated by averaging the stimulus intensity at all 40 measurement points.

In all the patients, OCT imaging was performed with Cirrus HD-OCT 5000 (ZEISS, Germany) with dimensions of 20° × 20° and a 47.2-µm 128-B-scan spacing. Each OCT scan obtained during each visit was independently assessed by two experienced physicians who were blinded to the patients’ clinical data. A third physician would be consulted if a disagreement arose. The test indicators included CRT, HRDs, EZ, and ELM. CRT was automatically generated by computer software. HRDs were defined as independent, dot-shaped lesions with equivalent or higher reflected signal strength than the retinal pigment epithelium (RPE) layer on an OCT scan^(16)^. The B-scan passing through the fovea was evaluated to determine the amount of HRD^(17)^. The number of HRDs in all retinal layers, entoretina (from the inner boundary membrane to the outer nuclear layer), ectoretina (from the ELM to the EZ), and subretinal layer (from the subretinal fluid to the RPE) were recorded. The number of HRDs was counted using a previously described method^(7)^; when the HRDs corresponded with the retinal hard exudates of FP, they were not counted. Disruptions of the ELM and EZ were defined as the horizontal extent with loss of the hyperreflective signal that characterizes each layer. FFA examination was performed using Spectralis HRA radiography (Heidelberg, Germany).

All the patients were followed up monthly for 6 months after the initial injection. At each visit, BCVA, mean retinal sensitivity, IOP measurement, and fundoscopy and OCT findings were assessed. In addition to these indicators, the number of injections, severe adverse reactions, and complications such as corneal edema, anterior chamber inflammation, cataract, active bleeding, retinal detachment, and severe ischemia were also recorded.

According to their BCVAs before and 6 months after treatment, the patients with improved visual acuity were included in the efficacy group; and those with no improvement in visual acuity and those with decreased visual acuity, in the inefficacy group.

### Statistical analyses

All statistical analyses were performed using SPSS Version 25.0 (SPSS, Inc., Chicago, IL, USA). All p values were two-sided with statistical significance at a level of 0.05. The intergroup comparison of the measurement data (presented as mean ± standard deviation) was analyzed using the independent sample *t* test, and the intragroup comparison was analyzed using the paired sample *t* test. Qualitative data (expressed as frequency percentage) were compared among the groups using the χ^2^ test. Variables with p values <0.1 in the univariate analyses were evaluated using a multivariate logistic regression analysis.

## RESULT

### Demographic data

A total of 58 eyes from 58 patients (31 women and 27 men, age: 70.07 ± 8.53 years) met the inclusion criteria. The baseline characteristics are summarized in table 1. Briefly, the mean IOP, duration of the disease course, BCVA, and CRT were 16.41 ± 2.88 mmHg, 6.90 ± 5.25 months, 1.10 ± 0.42 logMAR, and 440.38 ± 61.05 µm. The numbers of HRDs were 13.20 ± 6.04 (total), 4.76 ± 2.94 (entoretina), 2.97 ± 2.05 (ectoretina), and 5.46 ± 4.18 (subretinal layer), respectively. Eighteen patients had an intact ELM, and 23 patients had an intact EZ.

**Table 1:** Baseline characteristics of the patients with nAMD

Parameters	p Value
Patients, n (%)	58 (100%)
Age, years (mean±SD)	70.07±8.53
Sex (male/female, n/%)	27 (46.6%)/31 (53.4%)
Disease course, months (mean±SD)	6.90 ± 5.25
Baseline BCVA, logMAR (mean±SD)	1.10 ± 0.42
CRT,µm (mean±SD)	440.38 ± 61.05
Number of HRDs	
Total (mean±SD)	13.20 ± 6.04
Entoretina (mean±SD)	4.76 ± 2.94
Ectoretina (mean±SD)	2.97 ± 2.05
Subretinal layer (mean±SD)	5.46 ± 4.18
ELM	
Integrity, n (%)	18 (31.0%)
Disruption, n (%)	40 (69.0%)
EZ	
Integrity, n (%)	23 (39.7%)
Disruption, n (%)	35 (60.3%)
IOP, mmHg (mean±SD)	16.41 ± 2.88

BCVA= best-corrected visual acuity; nAMD= neovascular age-related macular degeneration; logMAR= logarithm of the minimum angle of resolution; CRT= central retinal thickness; HRD= hyperreflective dot; ELM= external limiting membrane; EZ= ellipsoid zone; IOP= intraocular pressure; SD= standard deviation.

### Treatment outcomes

After 6-month treatment, the mean BCVA improved from 1.10 ± 0.42 logMAR to 0.41 ± 0.18 logMAR, the mean retinal sensitivity increased from 5.13 ± 0.86 dB to 7.32 ± 1.21 dB, the mean CRT measurements decreased from 440.38 ± 61.05 µm to 260.01 ± 24.86 µm (all p<0.05; Table 2). From baseline to month 6, the total number of HRDs decreased from 13.20 ± 6.04 to 5.67 ± 4.01. The numbers of HRDs in the entoretina, ectoretina, and subretinal layer decreased from 4.76 ± 2.94 to 1.71 ± 1.95, from 2.97 ± 2.05 to 1.13 ± 1.47, and from 5.46 ± 4.18 to 2.84 ± 2.16 (all p<0.05; Table 2).

**Table 2:** Changes in BCVA, mean retinal sensitivity, CRT, and number of HRDs before and after treatment

Parameters	Baseline	M1	M2	M3	M4	M5	M6
BCVA (logMAR, mean±SD)	1.10 ± 0.42	0.76 ± 0.28*	0.61 ± 0.25*	0.52 ± 0.32*	0.47 ± 0.25*	0.42 ± 0.22*	0.41 ± 0.18*
Mean retinal sensitivity (dB, mean±SD)	5.13 ± 0.86	6.1 ± 0.71*	6.6 ± 0.69*	7.14 ± 0.76*	7.2 ± 0.83*	7.25 ± 0.75*	7.32 ± 1.21*
CRT (µm, mean±SD)	440.38 ± 61.05	310.12 ± 47.78*	297.91 ± 43.33*	267.82 ± 42.22*	272.26 ± 43.03*	275.34 ± 44.64*	260.01 ± 24.86*
Number of HRD							
Total (mean±SD)	13.20 ± 6.04	9.04 ± 5.91*	8.14 ± 5.06*	6.47 ± 4.81*	5.84 ± 4.26*	5.73 ± 4.17*	5.67 ± 4.01*
Entoretina (mean±SD)	4.76 ± 2.94	2.93 ± 2.63*	2.17 ± 2.03*	1.83 ± 1.96*	1.81 ± 1.74*	1.75 ± 1.88*	1.71 ± 1.95*
Ectoretina (mean±SD)	2.97 ± 2.05	2.03 ± 2.11*	1.67 ± 1.65*	1.26 ± 1.52*	1.23 ± 1.27*	1.17 ± 1.35*	1.13 ± 1.47*
Subretinal layer (mean±SD)	5.46 ± 4.18	3.83 ± 3.17*	3.24 ± 3.06*	2.94 ± 2.67*	2.85 ± 2.31*	2.79 ± 2.58*	2.84 ± 2.16*

BCVA= best-corrected visual acuity; logMAR= logarithm of the minimum angle of resolution; CRT= central retinal thickness; HRD= hyperreflective dot; SD= standard deviation; ^*^vs. baseline, p<0.05; M= month.

**Figure 1:**
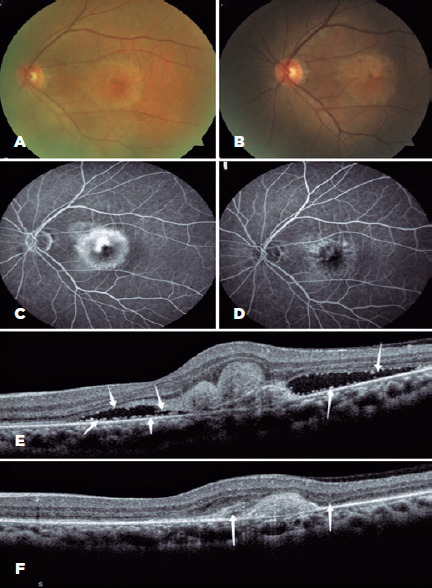
Representative fundus photochromy (FP), fundus fluorescein angiography, and optical coherence tomography images. (A) FP image before treatment showing circular raised foci in the macular area. (B) FP image at 6 months showing a smaller lesion in the macular area and less edema. (C) FFA image before treatment showing circular strong fluorescent areas with clear boundaries in the macular area. (D) FFA image at 6 months showing the reduction of fluorescence leakage in the macular area, weakened intensity, and a clear boundary. (E) OCT image before treatment showing neovascularization below the fovea, serious detachment of the neuroepithelium and retina pigment epithelium, and abundant HRDs in the subretinal layer. (F) OCT at 6 months image showing significant absorption of the subretinal HRD.

Twenty-two patients were included in the efficacy group; and 36 patients, in the inefficacy group. Figure 1 shows the results of fundus photography, FFA, and OCT of a representative patient in the efficacy group before and after treatment. In general, the treatment was effective, and no obvious abnormalities were observed. Subconjunctival hemorrhage occurred in 8 patients after IVC injection, which returned to normal 7 days after treatment. Four patients with elevated IOP returned to normal 3 days after treatment. During the follow-up period, no serious ocular complications related to treatment occurred, such as retinal detachment, retinal tear, continuous increase in IOP, and intraocular inflammation, as well as any serious systemic adverse reactions.

FP= fundus photochromy; FFA= fundus fluorescein angiography; OCT= optical coherence tomography; HRD= hyperreflective dots. The white arrows represent the HRDs.

### Independent predictors analysis

We found statistically significant differences between the efficacy and inefficacy groups in terms of age (p<0.001), baseline BCVA (p<0.001), total number of HRDs (p<0.001), number of HRDs in the subretinal layer (p<0.001), and ELM state (p=0.003). These variables, which showed statistically significant differences, were included in the multivariate logistic regression analysis. Age (OR= 1.198, 95% confidence interval [CI]: 1.005-1.427, p=0.044), baseline BCVA (OR=326.448, 95% CI: 2.218-48042.170, p=0.023), number of HRD in the subretinal layer (OR= 1.771; 95% CI: 1.202-2.610, p=0.004), and ELM status (OR=104.786, 95% CI: 2.321-4730.610, p=0.017) were independent predictors of the efficacy of IVC injection for patients with nAMD (Tables 3 and 4).

**Table 3:** Comparison of parameters between the efficacy and inefficacy groups

Parameters	Effiecacy group (n=22)	Inefficacy group (n=36)	p value
Age, years (mean±SD)	63.41 ± 9.78	74.14 ± 7.71	<0.001
Sex			0.896
Male, n (%)	10 (45.5%)	17 (47.2%)	
Female, n (%)	12 (54.5%)	19 (52.8%)	
Disease course, months (mean±SD)	6.46 ± 5.41	7.17 ± 5.23	0.140
Baseline BCVA, logMAR (mean±SD)	0.83 ± 0.33	1.26 ± 0.39	<0.001
Mean retinal sensitivity (dB, mean±SD)	4.97 ± 0.83	3.36 ± 0.64	0.108
CRT, µm (mean±SD)	427.97 ± 70.25	454.17 ± 75.31	0.193
Number of HRD			
Total (mean±SD)	9.23 ± 4.07	15.62 ± 5.79	<0.001
Entoretina (mean±SD)	3.77 ± 2.24	5.36 ± 4.16	0.105
Ectoretina (mean±SD)	2.42 ± 1.96	3.32 ± 2.62	0.170
Subretinal layer (mean±SD)	3.04 ± 2.05	6.94 ± 4.27	<0.001
ELM			0.003
Integrity, n (%)	12 (54.5%)	6 (16.7%)	
Disruption, n (%)	10 (45.5%)	30 (83.3%)	
EZ			0.208
Integrity, n (%)	11 (50%)	12 (33.3%)	
Disruption, n (%)	11 (50%)	24 (66.7%)	
IOP (mean±SD)	16.14 ± 3.09	16.57 ± 2.78	0.586

BCVA= best-corrected visual acuity; logMAR= logarithm of the minimum angle of resolution; CRT= central retinal thickness; HRD= hyperreflective dot; ELM= external limiting membrane; EZ= ellipsoid zone; IOP= intraocular pressure; SD= standard deviation. P values <0.05 were considered statistically significant.

**Table 4:** Univariate and Multivariate analyses of baseline characteristics and short-term visual acuity

Parameter	Univariate analysis		Multivariate analysis	
	OR (95% CI)	p value	OR (95% CI)	p value
Age	1.158 (1.067-1.258)	<0.001	1.198 (1.005-1.427)	0.044
Sex	0.931 (0.321-2.699)	0.896	-	-
Course of disease	1.058 (0.943-1.188)	0.140	-	-
Baseline BCVA	26.165 (3.949-173.371)	<0.001	326.448 (2.218-48042.170)	0.023
Mean retinal sensitivity	0.071 (0.003-1.789)	0.108	-	-
CRT	1.006 (0.998-1.014)	0.193	-	-
Number of HRDs				
Total	1.318 (1.127-1.541)	<0.001	1.665(0.793-3.499)	0.178
Entoretina	1.194 (0.993-1.435)	0.105	-	-
Ectoretina	1.262 (0.883-1.804)	0.170	-	-
Subretinal layer	1.703 (1.251-2.318)	<0.001	1.771 (1.202-2.610)	0.004
State of ELM	6.01 (1.783-20.191)	0.003	104.786 (2.321-4730.610)	0.017
State of EZ	0.500 (0.169-1.481)	0.208	-	-
IOP	1.015 (0.845-1.220)	0.586	-	-

BCVA= best=corrected visual acuity; LogMAR= logarithm of the minimum angle of resolution; CRT= central retinal thickness; HRD= hyperreflective dot; ELM= external limiting membrane; EZ= ellipsoid zone; IOP= intraocular pressure; OR= odds ratio; CI= confidence interval; NA, not applicable. P values <0.05 were considered statistically significant. Parameters with p values <0.1 in the univariate analysis were introduced in the multivariate analysis as independent variables.

## DISCUSSION

Our results demonstrated that baseline indicators such as the number of subretinal HRDs, ELM state, BCVA, and age were independent predictors of visual outcome at 6 months in the eyes with nAMD after IVC injection, which might have prognostic value for short-term visual outcomes.

In our study, IVC injection for nAMD showed certain efficacy and safety, consistent with the findings of many previous studies^(7,14,15)^. The indexes of BCVA, HRDs, and CRT were significantly improved after 6 months of IVC injection as compared with those before treatment. Although some patients had subconjunctival hemorrhage and transient IOP after increased treatment, they were all relieved spontaneously without serious adverse consequences and complications. Although the overall efficacy of conbercept was improved, the treatment effect was not so ideal for the individual patients. Thirty-six of the 58 patients had no improvement in vision after 6 months, which we speculated to be related to the severity of the disease reflected by the disease-related index before treatment. Understanding the relationship between baseline indicators and final visual acuity will be beneficial to the treatment and prognosis of patients.

Our study found that the number of subretinal HRDs at baseline was an independent predictor of BVCA at 6 months in the multiple logistic regression analysis. Moreover, the total number of HRDs and number of HRDs in the subretinal layer in the inefficacy group were significantly higher than those in the efficacy group, consistent with the previous studies in which initial presence of HRD in the foveal neurosensory retina was associated with poor final visual acuity^(18)^. Tang et al. demonstrated that the number of HRDs at baseline could be a good predictor of short-term visual outcome^(9)^. HRD proliferation and migration may serve as biomarkers for AMD progression. Some related mechanism research suggested that HRDs may originate from the RPE and may represent the migration of activated RPE cells to the inner layer of the retina in eyes with AMD^(19)^. Some laboratory studies also showed that RPE cells, induced by cytokines and other inflammatory mediators, may migrate in the presence of oxidative damage and complement activation^(20)^. We speculated that eyes with larger numbers of HRD corresponded with worse inflammatory response, resulting in a poorer visual outcome.

We observed that the state of ELM at baseline was also an independent predictor of BCVA at 6 months after treatment in the multivariate analysis. The ELM, a marker of photoreceptor function, was considered the zonula adherens between Müller cells and photoreceptors, and the potential for visual function and recovery may be directly assessed on the basis of its status^(21,22)^. Disruptive ELM could not stop extravasated lipoproteins from migrating and depositing in the outer layer of the retina, which may damage the photoreceptor status and Müller cells, causing poor vision^(23,24)^. Landa found that the integrity of the ELM layer appeared to be a critical factor for the restoration of the photoreceptor layer and for predicting a successful visual outcome^(25)^. At baseline, 12 patients (54.5%) in the efficacy group and 6 patients (16.7%) in the inefficacy group had an intact ELM, with statistically significant differences, suggesting that the final visual acuity prognosis of the patients with an intact ELM was relatively good. Similarly, some studies suggested that the intact initial ELM predicts a better visual outcome^(26)^.

Baseline BCVA was an important and independent predictor of all visual outcomes after 1- and 5-year anti-VEGF therapy. Generally, a better baseline BCVA predicted a better final BCVA^(27)^. Results from several real-world clinical trials have also shown that baseline BCVA was the strongest predictor of visual outcome^(28,29)^. In accordance with these results, we found that the final BCVAs of the patients were significantly lower than those before treatment, and the baseline BVCA in the efficacy group was lower than that in the inefficacy group, which suggests that good baseline visual acuity predicted better outcome. Age was also shown to be an important independent predictor of vision prognosis, and its importance has been confirmed in relevant studies^(8)^. In our study, the mean age in the efficacy group was 63.41 ± 9.78 years, which was significantly lower than that in the efficacy group (74.14 ± 7.71 years). This is not surprising because structural damage to the retinal structure and age-related functional decline in older patients may limit recovery potential.

We found, in agreement with previous studies, that sex had no impact on visual acuity end points^(28,29)^. Some studies found that the thicker the CRT, the worse the visual recovery after treatment, which suggests that baseline CRT may be a predictor of visual prognosis^(30)^. A recent study demonstrated that the extent of EZ disruption at baseline and its changes over time were associated with BCVA improvement at 3 months in a multivariate analysis, and a better integrity of EZ at baseline predicted a better BCVA^(9)^. Our study failed to identify an association between baseline CRT, baseline EZ, and BVCA at 6 months. The present study has some differences from the previous studies, which may be due to the small sample size and short follow-up observation time, so further research is needed to confirm the findings.

The limitations of our study were mainly due to its single-center retrospective design and small sample size, which might have limited our conclusions to some extent. Moreover, the inflammation after the injections was not evaluated. Therefore, further research with a more representative multicenter and large sample size is needed to examine the predictors of visual prognosis after IVC injection for nAMD.

The absence of a significant improvement in vision after IVC injection of conbercept in some patients with nAMD may be related to the number of subretinal HRDs, the state of ELM, baseline BCVA, and age, which is worthy of clinical attention. For patients with nAMD treated with IVC injection, the above-mentioned influencing factors should be fully evaluated before surgery. Identifying these predictors for good and poor visual outcomes is clinically relevant, as this information enables clinicians to better complement and improve clinical treatment for nAMD and provide advice on visual prognosis.
